# Splanchnic and cerebral oxygenation during cyclic phototherapy in preterm infants with hyperbilirubinemia

**DOI:** 10.1007/s00431-024-05810-1

**Published:** 2024-10-10

**Authors:** Carlo Dani, Giulia Remaschi, Federica Rossi, Monica Fusco, Chiara Poggi, Iuri Corsini, Simone Pratesi

**Affiliations:** 1 Department of Neurosciences, Psychology, Drug Research and Child Health, University of Florence, Florence, Italy; 2 Division of Neonatology, Careggi University Hospital of Florence, Largo Brambilla, 3, 50141 Florence, Italy

**Keywords:** Cyclic phototherapy, Splanchnic oxygenation, Near-infrared spectroscopy, Feeding intolerance

## Abstract

Cyclic phototherapy (cPT) can achieve a reduction in total serum bilirubin comparable to that achieved with standard continuous PT in preterm infants. Our aim was to assess the effect of cPT on splanchnic (rSO_2_S) and cerebral (rSO_2_C) oxygenation measured by near-infrared spectroscopy (NIRS). We prospectively studied 16 infants with a gestational age of 25–34 weeks with hyperbilirubinemia requiring PT. Splanchnic regional oxygenation (rSO_2_S), oxygen extraction fraction (FOES), and cerebro-splanchnic oxygenation ratio (CSOR) were recorded before, during, and after cPT discontinuation. We found that rSO_2_S, FOES, and CSOR did not change during the study period. The overall duration of single or multiple courses of PT was 6.5 (6.0–13.5) h, but by cycling PT, the actual exposure was 3.0 (1.5–4.9) h. Twelve patients (75%) required 15 min/h cPT, and four (25%) required prolonging cPT to 30 min/h. None of the patients developed enteral feeding intolerance.

*Conclusions*: cPT treatment of hyperbilirubinemia in preterm infants does not affect splanchnic oxygenation or intestinal oxygen blood extraction, likely due to the short exposure to PT light, and it could contribute to decreasing the risk of feeding intolerance.

**What is Known:**

*• The assumption that phototherapy (PT) is innocuous with no serious adverse effects has been questioned.*

*• Cyclic phototherapy (cPT) can achieve a reduction in total serum bilirubin comparable to that achieved with standard continuous PT in preterm infants.*

**What is New:**

*• Splanchnic and cerebral oxygenation measured by near-infrared spectroscopy did not change during KMC.*

*• cPT can contribute to decrease the risk of feeding intolerance likely due to the short exposure to PT light.*

## Introduction

A high level of total serum bilirubin (TSB) can be neurotoxic to the central nervous system in term and preterm infants, causing acute bilirubin encephalopathy (ABE) and neurological kernicterus spectrum disorders (KSDs) [1]. Preterm infants are at increased risk of developing KSDs [2] as demonstrated by a recent Japanese epidemiological study reporting that the occurrence of KSDs is 1.8 per 1000 live births < 30 weeks of gestational age [3]. Therefore, recommendations have been made for preventing ABE and KSDs in the neonatal period by establishing TSB threshold values at which to start phototherapy (PT), the first-choice treatment of neonatal hyperbilirubinemia. Some national guidelines also indicate the TSB threshold values ​at which to start phototherapy in preterm infants of different gestational ages [4, 5]. This point is important because the frequency of PT in very preterm infants is high and ranges from > 95% in infants with a birth weight of < 1000 g to > 90% in infants with a birth weight between 1001 and 1500 g [6].

However, the assumption that this therapy is innocuous with no serious adverse effects for even the most immature babies has been questioned. In fact, a randomized trial demonstrated that aggressive PT increases the mortality in extremely preterm infants [7], and other studies report several adverse effects, such as photo-oxidative injury, lipid peroxidation, DNA damage, reduced splanchnic oxygenation, feeding intolerance, and hemolysis [8–20]. Moreover, it has been suggested that these adverse effects of PT might increase the risk of epilepsy [21, 22] and cancer [23–26] in survivors.

Therefore, new approaches have been sought that are able to reduce TSB by limiting the duration of exposure to PT and the associated risk of adverse effects. Consistently, Arnold et al. [27] have shown that limiting the exposure to PT to a few minutes per hour (cyclic PT, cPT) can achieve a reduction in TSB like that achieved with standard continuous PT since photoisomerization of bilirubin in subcutaneous tissue or blood vessels near the skin surface is rapid [28–33].

On these bases, we hypothesized that cPT can avoid or limit the decrease in splanchnic oxygenation that has been shown to occur in association with continuous PT^14^. To assess this hypothesis, we studied a cohort of preterm infants requiring PT for hyperbilirubinemia whose splanchnic (rSO_2_S) and cerebral (rSO_2_C) regional oxygenation were measured by NIRS during cPT treatment.

## Material and methods

### Patient population

This observational prospective study was carried out at the third-level neonatal intensive care unit (NICU) of Careggi University Hospital of Florence, Italy, after approval by the Tuscany Pediatrics Ethics Committee. Infants with gestational age of 25–34 weeks were enrolled in the study within the first 2 weeks of life, after written parental informed consent, if they were affected by hyperbilirubinemia requiring PT. Exclusion criteria were birth weight of < 10th percentile, major congenital malformations, chromosomal disorders, life-threatening infections, and cardiovascular instability requiring treatment with vasoactive drugs.

### Study design

Enrolled patients were continuously studied by NIRS (Root® Masimo Corporation, Irvine, CA, USA) for measurement of rSO_2_S and rSO_2_C starting 30 min before the beginning of cPT and ending 12 h after its interruption, with a sampling interval of 6 s. The rSO_2_ measurements obtained with the NIRS technique reflect a combination of intravascular oxygenated/deoxygenated venous, arterial, and capillary hemoglobin in a ratio of approximately 75:20:5 [34]. This ratio has only been validated for the brain, although it has been extrapolated to other human tissues.

### Cyclic phototherapy

TSB was measured at least daily in whole blood from a heel prick sample in a blood gas analyzer with a spectrophotometer module. Entry criteria to PT followed the recommendations of the Italian Society of Neonatology [37]. Cyclic PT initially lasted 15 min/h, and its duration could be prolonged to 30 min/h or 60 min/h based on TSB values (Table 1). TSB levels were checked every 6 h, and PT was discontinued when the TSB level was below the threshold value for treatment. We used blue light emitting diodes PT (NeoBlu, Natus Medical Inc., San Carlos, CA). Diodes emitted light at 400 to 500 nm wavelengths (peak emission at 466 nm), and the device was placed about 30 cm above the infant (suggested mean irradiance = 3.50 µW/cm^2^), as recommended by the manufacturers. Commercially available plug-in timers were used to provide cPT.

**Table 1:** Duration of cycled phototherapy in relationship to total serum bilirubin (TSB) values. The duration of cycled phototherapy depends on the extent of the increase in the TSB value

Values of TSB	Cycled phototherapy duration
Less than threshold	No phototherapy
Threshold + 0.1–1.0 mg/dL	15 min/h
Threshold + 1.1–1.9 mg/dL	30 min/h
Threshold + > 2.0 mg/dL	60 min/h

### NIRS measurements

A self-adhesive transducer that contains a light-emitting diode and two distant sensors was placed on the forehead and infra-umbilical abdomen region of patients [35]. All measurements were taken with infants in a supine position. During data recording, infants were mostly quiet or sleeping, and to reduce NIRS artifacts, the handling of patients during the study period was minimized.

Based on the measurements of rSO_2_S, rSO_2_C, and SpO_2_, we calculated the splanchnic (FOES) and cerebral (FOEC) fractional oxygen extraction ratio (FOE) [36], which is the difference between arterial SpO_2_ measured by pulse oximetry and rSO_2_ measured by NIRS [FOE = (SpO_2_ − rSO_2_)/SpO_2_]. This parameter reflects the balance between oxygen delivery and oxygen consumption. Therefore, an increase in FOE suggests an increase in the oxygen extraction by tissues, due to higher oxygen consumption in relation to oxygen delivery, while its decrease suggests less oxygen use in comparison with the supply [36].

We also calculated the cerebro-splanchnic oxygenation ratio (CSOR, rSO_2_S/rSO_2_C), the ratio of oxygen saturation of splanchnic versus cerebral tissue. Since cerebral perfusion is subject to autoregulation while splanchnic perfusion is not, CSOR is reduced when there is a diversion of blood flow toward the vital organs, while it remains unchanged under normal conditions [36].

All NIRS data were recorded 30 ± 10 (*T*_Before_) min before PT, 60 ± 20 (*T*_60m_), 180 ± 30 (*T*_180m_), 360 ± 30 (*T*_360m_) min, 180 ± 30 (*T*_after180m_), 360 ± 30 (*T*_after360m_) min, and 12 ± 1 (*T*_after12h_) h after PT interruption, along with SpO_2_. All patients were studied only once.

### Data collection

For each studied infant, we reported gestational age, birth weight, sex, type of delivery, Apgar’s score at 5 min of life, antenatal steroids, ABO and Rh blood type incompatibility, age and TSB at the start of the first course of cPT, TSB after 6 h and at the end of cPT, duration of cPT and actual exposure to PT, requirement of 15 or 30 min/h cPT, increase of TSB after the start of cPT and peak TSB in these patients, requirement of more than one course of cPT, type and amount of milk fed at beginning of the study period, feeding intolerance during the study period, occurrence of non-invasive [nasal continuous positive airway pressure (NCPAP); nasal intermittent mandatory ventilation (N-IMV)] and invasive respiratory supports [patient triggered ventilation (PTV); high frequency oscillatory ventilation (HFOV)] at the start of cPT and their duration, patent ductus arteriosus (PDA) requiring treatment at the start of cPT, intraventricular hemorrhage (IVH) > grade 3, necrotizing enterocolitis (NEC) requiring surgical treatment, bronchopulmonary dysplasia (BPD), mortality, and length of hospital stay. PDA was diagnosed by echocardiography. Feeding intolerance was defined as a feeding withholding or a decrease of > 6 h or did not increase for > 24 h during PT^14^. Bronchopulmonary dysplasia was defined as oxygen requirement at 36 weeks of postconceptional age [38]. Intraventricular hemorrhage and NEC were diagnosed following Papile’s [39] and Bell’s [40] criteria, respectively.

### Statistical analysis

A sample size of at least 16 infants was calculated to be adequate to detect a statistically significant change of 10% of rSO_2_S measured after the starting of cPT with an 80% power at *P* < 0.05.

Patients’ clinical characteristics were described as mean ± SD, rate and percentage, or median and interquartile range (IQR). For each NIRS variable (rSO_2_S, FOES, rSO_2_C, FOEC, CSOR), we calculated the mean (± SD) from selected 5-min periods which were chosen at the end of *T*_Before_, *T*_60m_, *T*_180m_, *T*_360m_, *T*_12h_, *T*_24h_, *T*_after180m_, *T*_after360m_, and *T*_after12h_ [35]. We made this choice to obtain the highest stability of the NIRS signal. However, sometimes, this was not possible due to the occurrence of unwanted artifacts (generally infant movements): in this case, the 5-min period without artifacts closest to the end of the study period was selected. Since our data were normally distributed, the serial measurements of studied variables were compared by repeated-measures analysis of variance (ANOVA). *P* < 0.05 was considered statistically significant.

## Results

### Clinical characteristics of studied infants (detailed in Table 2)

**Table 2:** Clinical characteristics and complications rate in studied infants. Mean (± SD), median (IQR), or number (%)

	*n* = 16
Gestational age (wks)	28.6 ± 2.8
Birth weight (g)	1174 ± 105
Female	9 (56)
Antenatal steroids	14 (88)
Vaginal delivery	11 (69)
Apgar score at 5 min	8 (8–8)
Volume of milk fed at the beginning of the study period (ml/kg)	69 ± 39
Human milk	14 (87)
Mixed milk	2 (13)
Feeding intolerance during the study period	0
Non-invasive ventilation	16 (100)
Mechanical ventilation	1 (6)
Respiratory distress syndrome	16 (100)
Patent ductus arteriosus	5 (31)
Bronchopulmonary dysplasia	3 (19)
Necrotizing enterocolitis	0
Intraventricular hemorrhage > grade 3	0
Death	0
Duration of hospital stay (*d*)	65 ± 18

Cyclic PT started at the median age of 62 (46–127) h of life. Twelve patients (75%) required 15 min/h cPT, and four (25%) required to prolong cPT to 30 min/h. TSB decreased from 9.2 (7.6–10.3) mg/dL at the start of cPT to 8.3 (7.7–9.0) mg/dL after 6 h of treatment and to 8.3 (7.1-–0.0) mg/dL at the end of cPT. In five (31%) patients, TSB increased after the start of cPT, and their peak TSB was 8.4 (8.1–9.4) mg/L. It is noteworthy that the overall duration of single or multiple courses of PT was 6.5 (6.0–13.5) h, but by cycling PT, the actual exposure was 3.0 (1.5–4.9) h (Table 3).

**Table 3:** Characteristics of cyclic phototherapy and total serum bilirubin (TSB) in studied infants. Median and (IQR) or numeber (%)

	*n* = 16
ABO and Rh blood type incompatibility	5 (31)
Age at the start of cyclic phototherapy (h)	62 (46–127)
15 min/h cyclic phototherapy	12 (75)
30 min/h cyclic phototherapy	4 (25)
TSB at the start of cyclic phototherapy (mg/dL)	9.2 (7.6–10.3)
TSB after 6 h of cyclic phototherapy (mg/dL)	8.3 (7.7–9.0)
TSB at the end of cyclic (mg/dL)	8.3 (7.1–9.0)
Increase of TSB after the start of cyclic phototherapy	5 (31)
Peak of TSB (mg/dL)	8.4 (8.1–9.4)
Duration of cyclic phototherapy (h)	6.5 (6.0–13.5)
Actual exposure to phototherapy (h)	3.0 (1.5–4.9)
> 1 course of phototherapy	4 (25)

**Fig. 1:**
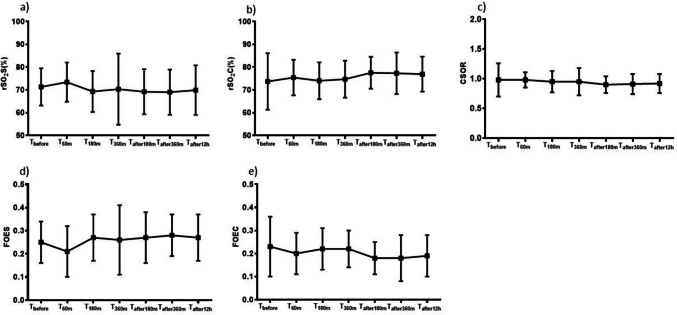
Changes of **a** splanchnic oxygenation (rSO_2_S), **b** cerebral oxygenation (rSO_2_C), **c** cerebro-splanchnic oxygenation ratio (CSOR), **d** splanchnic fractional oxygen extraction (FOES), and **e** cerebral fractional oxygen extraction (FOES) at the different data points of the study. Mean ± (SD)

The mean value of rSO_2_S was 70.3 ± 9.9%. It did not change (*P* = 0.824) during the study period ranging from 71.3 ± 8.2% at baseline to 69.9 ± 10.9% at *T*_after12h_ and reaching its lowest value at *T*_after360m_ (69.0 ± 9.9%). Similarly, FOES did not change (*P* = 0.995) during the study period, ranging from 0.25 ± 0.09 at baseline to 0.27 ± 0.10 at *T*_after12h_ and reaching its lowest value (0.21 ± 0.11) at *T*_60m_ (Table 4, Fig. 1).

**Table 4:** Changes of splanchnic (rSO_2_S) and cerebral (rSO_2_C) oxygenation, splanchnic (FOES) and cerebral (FOEC) fractional oxygen extraction ratio, and cerebro-splanchnic oxygenation ratio (CSOR) at the different data points of the study. Mean ± (SD)

	*T*_Before_	*T*_60m_	*T*_180m_	*T*_360m_	*T*_after180m_	*T*_after360m_	*T*_after12h_	*P*
rSO_2_S (%)	71,3 ± 8.2	73.4 ± 8.6	69.3 ± 9.0	70.3 ± 15.6	69.2 ± 9.9	69.0 ± 9.9	69.9 ± 10.9	0.824
FOES	0.25 ± 0.09	0.21 ± 0.11	0.27 ± 0.10	0.26 ± 0.15	0.27 ± 0.11	0.28 ± 0.09	0.27 ± 0.10	0.995
rSO_2_C (%)	73.7 ± 12.4	75.4 ± 7.8	74.0 ± 8.1	74.7 ± 8.1	77.5 ± 7.0	77.3 ± 9.1	76.9 ± 7.7	0.577
FOEC	0.23 ± 0.13	0.20 ± 0.09	0.22 ± 0.09	0.22 ± 0.08	0.18 ± 0.07	0.18 ± 0.10	0.19 ± 0.09	0.942
CSOR	0.98 ± 0.28	0.98 ± 0.13	0.95 ± 0.18	0.95 ± 0.23	0.90 ± 0.14	0.91 ± 0.17	0.92 ± 0.16	0.995

During the study period, the mean value of rSO_2_C was unchanged. The lowest value of rSO_2_C (74.0 ± 8.1%) was recorded at *T*_180m_. Similarly, FOEC did not significantly change (*P* = 0.942). The lowest value of FOEC (0.18 ± 0.07) was recorded at *T*_180m_. (Table 4, Fig. 1).

We observed that CSOR did not vary (*P* = 0.995) during or after cPT (Table 4, Fig. 1).

## Discussion

In this study, we assessed whether cPT avoids or limits the decrease (observed after continuous PT^14^) of splanchnic oxygenation in preterm infants with hyperbilirubinemia; if so, cyclic PT could reduce feeding intolerance associated with PT^15^. We found that rSO_2_S measured by NIRS did not change during or after cPT. The lack of changes in FOEC confirmed that intestinal oxygen blood extraction was not affected by cPT and that rebalancing mechanisms to counteract negative changes in oxygenation were not required. These findings are further supported by the stability of CSOR in our population, which suggests that cPT did not induce a diversion of blood flow from splanchnic to cerebral tissues as appears to occur during continuous PT^14^.

To explain our results, it is important to underline that in our patients, the actual exposure to phototherapy was only 3.0 (1.5–4.9) h, as compared to a total duration of the cPT cycle of 6.5 (6.0–13.0) h. Previous studies reported a negative effect of continuous PT on mesenteric blood flow-limiting intestinal perfusion and its increase post-feeding [41, 42]. Many mechanisms have been suggested to explain these detrimental hemodynamic effects, such as light-induced peripheral vasodilation mediated by nitric oxide (NO) and cyclic guanosine monophosphate (cGMP) pathway [43, 44]; a PT-induced increase of NO level not balanced by a proportional increase of endothelin (ET), possibly leading to a dilation of blood vessels [45]; a decrease in cardiac output followed by the activation of autonomic compensatory mechanisms, resulting in blood flow redistribution [43, 44]; and the diversion of blood flow to the skin, limiting the mesenteric perfusion [41, 46] and decreasing intestinal oxygenation. Therefore, it seems reasonable to affirm that the halving of light exposure that we achieved in our patients using cPT instead of traditional continuous PT may explain our findings.

Since the effects of PT can last hours after its discontinuation [41, 43, 44], we extended NIRS monitoring to 12 h after the end of cPT and found no changes in rSO_2_S. On the other hand, supporting the lack of effects of cPT on splanchnic hemodynamics, we also observed that none of the infants developed enteral feeding intolerance during cPT, while in a previous study, we found that 20% of patients developed it during continuous PT^14^.

We observed that PT was given for no more than 15 min/h to 75% of patients in agreement with Arnold et al. [27] who found a similar (82%) requirement in their infants treated with cPT. Furthermore, the duration of cPT was shorter (13.2 ± 7.4 vs. 34 ± 19 h) than previously reported [27], likely because our patients were less immature and their hyperbilirubinemia less severe and persistent. In any case, our data confirmed that cPT is effective in lowering TSB despite the lower actual exposure to PT since only 25% of infants required more than one course of PT and no patients required an exchange transfusion.

Limitations include the population size which did not allow accurate evaluation of the effect of cPT on enteral feeding tolerance and precluded the possibility of assessing possible differences in rSO_2_S between infants who were tolerant or non-tolerant of enteral nutrition.

In conclusion, our study demonstrates that cPT treatment of hyperbilirubinemia in preterm infants does not affect splanchnic oxygenation and intestinal oxygen blood extraction and could contribute to decreasing the risk of feeding intolerance [15]. This likely occurs because during cPT the actual exposure to PT light is too short to induce peripheral vasodilation and trigger a redistribution of blood flow which can limit mesenteric perfusion. These results may contribute to the spread of cPT which remains poorly diffused despite its efficacy [27] and the adverse effects of continuous PT.

## Data Availability

Data are available upon reasoned request.

## Abbreviations

cPT: Cyclic phototherapy
CSOR: Cerebro-splanchnic oxygenation ratio
FOEC: Cerebral tissue oxygen extraction fraction
FOEs: Splanchnic lung tissue oxygen extraction fraction
PT: Phototherapy
rSO_2_C: Cerebral oxygenation
rSO_2_S: Splanchnic oxygenation
TSB: Total serum bilirubin
